# Identification of functional SNPs in the 5-prime flanking sequences of human genes

**DOI:** 10.1186/1471-2164-6-18

**Published:** 2005-02-17

**Authors:** Salim Mottagui-Tabar, Mohammad A Faghihi, Yosuke Mizuno, Pär G Engström, Boris Lenhard, Wyeth W Wasserman, Claes Wahlestedt

**Affiliations:** 1 Center for Genomics and Bioinformatics, Karolinska Institutet, SE-17177 Stockholm, Sweden; 2 Centre for Molecular Medicine and Therapeutics, University of British Columbia, Vancouver, BC V5Z 4H4, Canada

## Abstract

**Background:**

Over 4 million single nucleotide polymorphisms (SNPs) are currently reported to exist within the human genome. Only a small fraction of these SNPs alter gene function or expression, and therefore might be associated with a cell phenotype. These functional SNPs are consequently important in understanding human health. Information related to functional SNPs in candidate disease genes is critical for cost effective genetic association studies, which attempt to understand the genetics of complex diseases like diabetes, Alzheimer's, etc. Robust methods for the identification of functional SNPs are therefore crucial. We report one such experimental approach.

**Results:**

Sequence conserved between mouse and human genomes, within 5 kilobases of the 5-prime end of 176 GPCR genes, were screened for SNPs. Sequences flanking these SNPs were scored for transcription factor binding sites. Allelic pairs resulting in a significant score difference were predicted to influence the binding of transcription factors (TFs). Ten such SNPs were selected for mobility shift assays (EMSA), resulting in 7 of them exhibiting a reproducible shift. The full-length promoter regions with 4 of the 7 SNPs were cloned in a *Luciferase *based plasmid reporter system. Two out of the 4 SNPs exhibited differential promoter activity in several human cell lines.

**Conclusions:**

We propose a method for effective selection of functional, regulatory SNPs that are located in evolutionary conserved 5-prime flanking regions (5'-FR) regions of human genes and influence the activity of the transcriptional regulatory region. Some SNPs behave differently in different cell types.

## Background

Single nucleotide polymorphisms (SNPs) are the most common form of genomic variations occurring on average every 1000 nucleotides. The vast majority of SNPs are neutral allelic variants, however the few that do influence a phenotype in a measurable way, are important for understanding the underlying genetics of human health. SNPs are the focus of a large number of human genetics studies attempting to understand their impact on complex diseases like Alzheimers, Parkinsons, diabetes, etc. Most SNPs, by the virtue of their location within genes (introns, 3'-UTRs, etc) or between genes, are considered most likely to be benign and not to contribute to a phenotype, whether it may be the manifestation of a disease or quicker metabolism of a drug. Among the group of SNPs located within coding regions of genes and causing a change in the peptide sequence (non-synonymous SNPs or 'nsSNPs') or among SNPs located within promoters (regulatory SNPs or rSNPs), a majority may not influence the overall activity of the protein or the gene expression. With the per-SNP validation and genotyping cost relatively high, it is increasingly important to develop strategies to predict functionally relevant SNPs *in silico*. The SNP databases in public domain, like NCBI/dbSNP and HGVbase, have facilitated this by highlighting all nsSNPs and also further classifying the location of the amino acid within the encoded proteins [1] to more accurately predict the detrimental effects of a change in peptide sequence. Several recent studies have attempted to focus on the subset of nsSNPs that most likely influence phenotype [2-6]. Of the approximately 4.5 Million SNPs in dbSNP [7], an estimated 10,000 nsSNP exist and approximately 10–15% of those are projected to be damaging [6]. Comparatively fewer attempts have been made to predict and validate functional promoter SNPs [8].

Transcriptional regulatory regions in the 5'-FR of human genes encode short (often < 25 bp) [9,10] sequences which serve as targets for binding of transcription factors (TFs). Understanding the conditions of binding, specificity and identity of the factors would help us understand the mechanism of regulation of human genes. Eukaryotic TFs tolerate considerable sequence variation in their target sites and recent bioinformatics works [11-13] have developed methods to model the DNA binding specificity of individual TFs [10]. Such matrices, although highly accurate [9,14], are less specific in the identification of sites with *in vivo *function [11], mainly due to our limited understanding of additional factors involved in TF specificity such as factor cooperative binding, protein-protein interactions, chromatin superstructures and TF concentrations. Currently the most successful approach to overcome this information gap is based on the assumption that sequences conserved between species (here human and mouse) would most likely mediate biological function [15-19].

The 7TM (7 trans-membrane domain proteins), also known as the hetero-trimeric GTP-binding protein (G protein)-coupled receptors (GPCRs) are members of a large family with an estimated 700 genes in the human genome [20]. By some estimates, nearly 60% of drugs marketed today target directly or indirectly the GPCR family members [21]. Several studies have collectively analyzed the occurrence, and importance, of coding SNPs to pharmaceutical efforts, in this family of genes [22-24]. Characterizing polymorphisms that are located in the 5'-FR of these genes and that influence expression has been reported earlier. We therefore selected a subset of clinically and pharmacologically important GPCR genes and their 5'-FR sequences to test our bioinformatics and laboratory experimentation approach for prediction of functionally important SNPs in regulatory regions. Our selection system evaluates the influence of SNPs on TF-DNA complex stability, and further investigates the influence of such SNPs on promoter activity. We present a proof-of-concept for such a strategy and identify issues and problem-areas for future developments.

## Results

**Figure 1:**
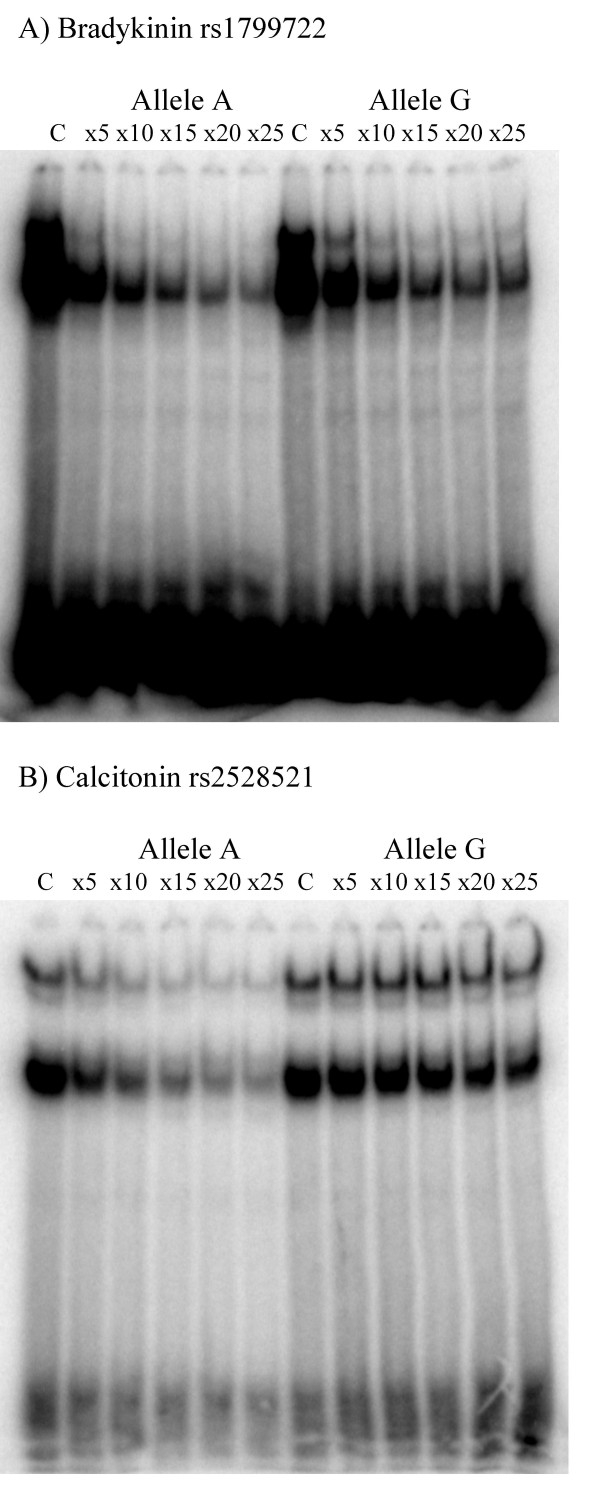
**Polyacrylamide gels from electromobility shift assays.**  Polyacrylamide gels showing the decrease in amounts of protein complex with labelled oligomer as the concentration of the competing non-labelled oligomer increases (lanes marked x5 through x25). Lane marked ’C’ has no competitor and represents the basal levels of labelled complex. The measure of displacement of the labelled oligomer is expressed as a ratio of  radio-labelled product, for each lane, divided by the basal value, presented in Table 2. Comparison of allele specific DNA-protein complex stability is a ratio of the highest competitor concentration (x25) of each of the two alleles. Thus for rs1799722 (A) allele A binds proteins 1.66 times better than allele G and for rs2528521 (B) allele A binds proteins 2.62 times better than allele G (Table 2, extreme right column).

The lists of the full names and Ensembl ENSG numbers [25] of the 176 GPCR genes are shown in additional files [See Additional file 1]. From a total of approximately 800 SNPs in proximal 5 kb regions, less than 200 were mapped to regions of mouse-human genome conservation. Of these approximately 200 SNPs, 36 were predicted to influence TF binding, in regions of sequence conservation of over 70% in human-mouse; the alignments for two such regions are indicated in additional files [See Additional file 2]. Table 1 lists the 21 genes, along with the SNPs, TFs and TFBS sequences and positions relative to transcription start site. These 36 candidate SNPs in Table 1 were qualified by our selection criteria, as described in the methods section, and were predicted to influence the binding of TFs in a qualitative manner. The absolute binding score of the TF differed by at least 2 units between the two alleles. Ten SNPs within the 5'-FR of 7 genes were selected for EMSA tests and are shown in bold letters in Table 1. The choice of these genes was based on our understanding of their significance in human physiology and relevance to research interests within the GPCR research community. Table 2 shows the results from the EMSA experiments, where values in each column for Allele 1 and Allele 2 are the ratios of measurements from each of the 5 different concentrations of the competitor oligomers (labeled × 5 through × 25 in Table 2 and in Figure 1) divided by the measurements without competitor (labeled 'C' in Figure 1). The decrease in level of the labeled product as a consequence of increasing non-labeled oligomer concentration is an indication of the efficiency of displacement, thereby reflecting the relative stability of the DNA-protein complex. A marginal increase in level of radio-labeled complex, instead of a decrease (Table 2 rs1800508) is generally considered to be due to additional factor involvements. Table 2, column 'Ratio' shows the difference, calculated for the highest concentration of the non-labeled competitor (25-fold), between the efficiency of competition between a perfect-match competitor and the allelic mismatch competitor. Values close to 1.00 indicate no difference in rate of competition between the two alleles, and therefore no relative difference in stability of the DNA-protein complex. While 4 SNPs exhibit mobility shift with a difference of 2-fold or more (rs267412, rs509813, rs945032 and rs2528521), three SNPs exhibit a moderate, nevertheless reproducible shift (rs1799722, rs2882225 and rs1538251). Finally, three SNPs fail to show any significant shift (rs968554, rs267413 and rs1800508). In all, seven out of ten polymorphic markers indicated reproducible binding differences between the alleles. Figure 1 shows pictures of EMSA gels for two of the SNPs, rs1799722 and rs2528521.

**Table 1:** List of positive SNP candidates for EMSA studies. The genes and the SNPs which were tested in this report are indicated in bold text. The name of the TF and the predicted consensus site from the transcription start site are indicated.

Gene Name /Ensembl ENSG	TF name (distance from start site)	SNP rs ID	Allele 1	Allele 2
Beta-2 adrenergic receptor ENSG00000164272	Nkx(-4257)	rs2082382	ttcagtg	ttcggtg
	Gklf(-4216)	rs2082395	aagtgagaag	aagtgagaaa
	c-ETS(-1049)	rs1432622	gatcct	gatctt
P2y purinoceptor 5 (p2y5) ENSG00000139679	SAP-1(-149)	rs2233571	agcggaaat	agtggaaat
Anion exchange protein 2 ENSG00000164889	Yin-Yang(-3480)	rs2069453	gccatg	gccgtg
	c-ETS(-2988)	rs2069451	tttccc	tgtccc
	SPI-1(-2988)	rs2069451	gggaaa	gggaca
	SPI-B(-2990)	rs2069451	atgggaa	atgggac
	c-MYB_1(-1536)	rs2069442	gggagttg	gggacttg
	Nkx(-1537)	rs2069442	tcaagtc	tcaactc
	SP1(-1440)	rs2069441	agggctggga	agagctggga
C-c chemokine receptor type 1 ENSG00000163823	SPI-B(-117)	rs3181080	acaagaa	actagaa
	SOX17(-118)	rs3181080	tttcttgtc	tttctagtc
Frizzled 6 precursor (frizzled-6) ENSG00000164930	deltaEF1(-629)	rs3758096	caccta	aaccta
Proteinase activated receptor 3 ENSG00000164220	c-ETS(-32)	rs2069647	catcct	cctcct
	deltaEF1(-31)	rs2069647	ctcctt	atcctt
Chemokine (C-X-C) receptor 6 ENSG00000163819	c-MYB_1(-469)	rs2234352	tacagatg	tatagatg
	Thing1-E47(-469)	rs2234352	catctgtaaa	catctataaa
5-hydroxytryptamine 1a receptor **ENSG00000178394**	ARNT(-2174)	**rs968554**	caagtg	caactg
	c-MYB_1(-2174)	**rs968554**	tccagttg	tccacttg
	deltaEF1(-2174)	**rs968554**	cacttg	cagttg
	n-MYC(-2174)	**rs968554**	cacttg	cagttg
	USF(-2174)	**rs968554**	caagtgg	caactgg
	USF(-2175)	**rs968554**	cacttgg	cagttgg
	ARNT(-2174)	**rs968554**	cacttg	cagttg
	deltaEF1(-2174)	**rs968554**	caactg	caagtg
	n-MYC(-2174)	**rs968554**	caagtg	caactg
Muscarinic acetylcholine receptor M1 **ENSG00000168539**	MZF_1-4(-148)	**rs509813**	tggggg	tggcgg
	MZF_5-13(-149)	**rs509813**	gtggggggag	gtggcgggag
	MZF_1-4(-147)	**rs509813**	gggggg	ggcggg
	SP1(-147)	**rs509813**	ggggggagga	ggcgggagga
Dopamine receptor D1a **ENSG00000184845**	FREAC-4(-4311)	**rs267412**	gtaaaccc	gtaagccc
	TCF11MafG(-4446)	**rs267413**	actgac	acagac
Follicle stimulating hormone receptor **ENSG00000170820**	HFH-3(-81)	**rs2882225**	ggatgctttttt	ggatgctgtttt
	HFH-2(-82)	**rs2882225**	gatgcttttttt	gatgctgttttt
	c-ETS(-80)	rs2349718	cttctt	cttttt
	Gklf(-87)	rs2349718	aaaaaaaaag	aaaaaaaaaa
	SPI-1(-80)	rs2349718	aagaag	aaaaag
5-hydroxytryptamine 2c receptor ENSG00000147246	HFH-1(-1736)	rs3795182	ccatgtttata	ccatatttata
	MEF2(-1734)	rs3795182	atatttataa	atgtttataa
	FREAC-4(-1734)	rs3795182	ataaacat	ataaatat
	c-ETS(-271)	rs3813928	tatcct	taccct
	MZF_1-4(-273)	rs3813928	tgagga	tgaggg
	SPI-B(-273)	rs3813928	tgaggat	tgagggt
Bradykinin receptor 2 **ENSG00000168398**	Ahr-ARNT(-535)	**rs945032**	tgggtg	tgggta
	MZF_1-4(-80)	**rs1800508**	tgggca	tgagca
	AP2alpha(-79)	**rs1800508**	gcccaggag	gctcaggag
	TCF11-MafG(-61)	**rs1799722**	aatgat	agtgat
Adenosine A3 receptor **ENSG00000121933**	AP2alpha(-4276)	**rs1538251**	gccctctgg	tccctctgg
Alpha-1a adrenergic receptor ENSG00000120907	c-ETS(-4898)	rs562843	cttctt	cttatt
	SPI-1(-4898)	rs562843	aagaag	aataag
	Nkx(-4900)	rs562843	ataagtt	agaagtt
C-c chemokine receptor type 2 ENSG00000121807	TCF11MafG(-1823)	rs3092964	catgcc	catacc
	Ahr-ARNT(-1825)	rs3092964	tgcatg	tgcata
	TCF11MafG(-1823)	rs3749462	catgcc	catacc
	Ahr-ARNT(-1825)	rs3749462	tgcatg	tgcata
Putative chemokine receptor ENSG00000119594	SPI-B(-86)	rs3825163	tcaggaa	ccaggaa
	FREAC-4(-80)	rs3825163	gtaaccat	ataaccat
	TCF11-MafG(-81)	rs3825163	gataac	ggtaac
	Nkx(-161)	rs2256572	ttatttg	ctatttg
	S8(-163)	rs2256572	tatta	tacta
	TCF11MafG(-232)	rs590447	gctgac	gccgac
	deltaEF1(-230)	rs590447	cagctt	cggctt
Calcitonin receptor **ENSG00000004948**	TCF11MafG(-511)	**rs2528521**	agtgac	agtggc
Lectomedin-3 ENSG00000150471	AP2alpha(-770)	rs905963	gccccgagc	accccgagc
	SPI-1(-763)	rs905963	gcgaac	gcgagc
	c-ETS(-365)	rs1505666	cctcct	ccttct
	SPI-1(-366)	rs1505666	gagaag	gaggag
	MZF_1-4(-367)	rs1505666	agagga	agagaa
Glucagon-like P2 ENSG00000065325	SPI-B(-881)	rs1402655	tgagaaa	tgataaa
G protein-coupled receptor ENSG00000102865	Yin-Yang(-118)	rs2240047	gccatg	gccctg
	TCF11MafG(-118)	rs2240047	catggc	cagggc
	Gfi(-1575)	rs724615	aaaatcacag	aaaatgacag

**Table 2:** Oligonucleotide sequences used for EMSA. For every SNP, 4 oligonucleotides (2 complimentary pairs) were synthesized, one pair for each allele. One oligonucleotide sequence from each pair had additional GG dinucleotide overhangs at the 5'end for fill-in labeling reaction. Care was taken to make sure that the additional GG-dinucleotide did not influence the predicted TF binding capability. The complementary sequences lacked the GG pairs. Only the allelic sequence predicted to bind most stably was chosen for the fill-in labeling reaction (marked *) while the gel shift assays were carried out using competitor with a perfect match versus a competitor with the allelic mismatch. The polymorphic site is underlined. Column 'Ratio' shows the difference in competition between the labeled and non-labeled oligomers at 25-fold excess, by dividing Allele2 (x25) values by Allele1 (x25).

Gene name and rs ID	Sequence	Allele 1(competitor oligo is a perfect match)					Allele 2 (competitor oligo has a mismatch)					Ratio
		x5	x10	x15	x20	x25	x5	x10	x15	x20	x25	
Serotonin receptor (5-HT-1A) rs968554 ENST00000323865	GGAAAAGAATCCA CTTGGGCCAATG * GGAAAAGAATCCA GTTGGGCCAATG	1.29	-	1.27	1.23	1.00	1.16	-	1.12	1.13	0.96	0.96
Dopamine receptor DRD1 rs267412 ENST00000329144	GGAATGTAAACCC AACACAAAAG * GGAATGTAAGCCC AACACAAAAG	0.72	-	-	0.54	0.56	0.98	-	1.06	1.03	1.12	2.05
Dopamine receptor DRD1 rs267413 ENST00000329144	GGTATAAAAGTCA GTGAATACAG * GGTATAAAAGTCT GTGAATACAG	0.96	-	0.81	0.74	0.81	0.97	-	0.98	0.97	1.01	1.24
Muscarinic acetylcholine receptor M1 rs509813 ENST00000306960	GGCTTGGGCTCCT CCCCCCAGCCAAC * GGCTTGGGCTCCT CCCGCCAGCCAAC	0.21	0.11	0.08	0.05	0.09	0.70	0.60	0.46	0.46	0.37	4.11
Follicle stimulating hormone receptor. rs2882225 ENST00000304421	GGCAAGGGAGCTG TTTTTTTT GGCAAGGGAGCTT TTTTTTTT *	1.10	1.00	0.86	0.71	0.75	1.96	1.73	1.67	1.36	1.35	1.80
Adenosine-A3 receptor. rs1538251 ENST00000241356	GGTGGCCACCAGA GGGCAGCACG * GGTGGCCACCAGA GGGAAGCACG	1.08	1.11	0.95	0.89	0.76	1.49	1.41	1.44	1.51	1.40	1.84
Bradykinin receptor B2 rs1800508 ENST00000306005	GGGAAGTGCCCAG GAGGC * GGGAAGTGCTCAG GAGGC	1.67	1.28	1.17	1.16	1.20	1.10	1.06	1.11	1.27	1.33	1.03
Bradykinin receptor B2 rs945032 ENST00000306005	GGTTCCTGGGTGC GGG * GGTTCCTGGGTAC GGG	0.88	0.72	0.72	0.62	0.55	1.15	1.22	1.29	1.22	1.27	2.30
Bradykinin receptor B2 rs1799722 ENST00000306005	GGCTGGGTAGTGA TGTCATCAGC GGCTGGGTAATGA TGTCATCAGC *	0.36	0.21	0.16	0.12	0.12	0.50	0.29	0.22	0.20	0.20	1.66
Calcitonin receptor precursor rs2528521 ENST00000316576, ENST00000248548	GGCTGTCCCCGGA GTGGCGGCT GGCTGTCCCCGGA GTGACGGCT *	0.54	0.36	0.25	0.22	0.21	0.90	0.83	0.73	0.60	0.55	2.62

For EMSA-positive markers with no validation information at dbSNP, HGVbase or Celera Discovery Systems™ we did a validation analysis using RFLP (restriction fragment length polymorphism) on DNA samples from 25 healthy Nordic individuals. The three SNPs which failed to show positive gel-shift results (serotonin receptor 5HT-1A: rs968554; DRD1: rs267413; and BK-2: rs1800508) were not investigated any further. For dopamine receptor D1 (DRD1) polymorphism (rs267412) the genotype distribution was found to be TT = 30%, AA = 20% and AT = 50%, and for calcitonin receptor promoter (CT-R) polymorphism rs2528521, it was GG = 40%, AA = 30% and GA = 30%. While rs267412 was found to be in Hardy Weinberg Equilibrium (HWE), rs2528521 was not. A larger population sample should be genotyped to accurately measure HWE for both these loci. Bradykinin B2 (BK-2) promoter polymorphism rs1799722 is noted to be polymorphic (major allele C: 56%) at NCBI/dbSNP. The polymorphic nature of this locus (rs1799722) was also confirmed by crosschecking at Celera Discovery Systems™. Also for the second BK-2 SNP rs945032, the allele frequency information was found at dbSNP (major allele = 80%). NCBI's dbSNP provided no allele frequency information about rs1538251 adenosine-A3 receptor (ADORA3), and on sequencing 20 DNA samples this marker proved to not be polymorphic in Nordic sample population, and was eliminated from further analysis (data not shown). The genotype frequency of muscarinic acetylcholine receptor M1 (CHRM1) SNP rs509813 was documented at Celera Discovery Systems™. The contig-position of rs2882225 (follicle simulating hormone receptor; FSHR) was not in agreement between the three major public genome databases, i.e. NCBI, Ensembl and Santa Cruz Genome Assembly (UCSC). It was mapped within the transcript for FSHR by NCBI/dbSNP, and completely absent from Ensembl and UCSC [26]. Therefore of the seven SNPs exhibiting positive EMSA, five SNPs (in four genes) qualified for analysis of their influence on promoter activity.

**Figure 2:**
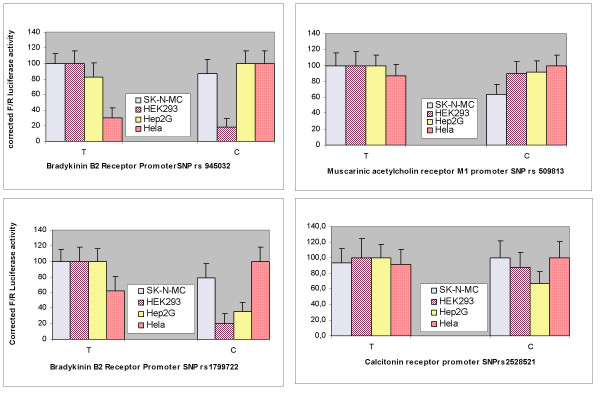
**Comparative promoter activity in different cell lines.**  Influence of four functional promoter SNPs on promoter activity is dependent on cell types. Measurements are an average of four independent experiments. A ‘T’ indicates an ‘AA’ and a ‘C’ indicates a ‘GG’ genotype.

For expression analysis in living cells, the published promoter regions, or putative regulatory 5'FR of up to 2 Kb, were cloned and basal levels of luciferase were monitored. Repeated attempts to clone the promoter region of DRD1 failed (data not shown). Furthermore the position of rs267413 is mapped at -4446 nucleotides with respect to the transcription start site, whereas the minimum length of the genomic fragment known to drive DRD1 expression is limited to 2571 nucleotides. Considering the distal position of the marker, we decided not to examine rs267413 further in this study. The promoter regions of BK-2, CHRM1 and CT-R were cloned successfully. A total of four dissimilar human cell lines (HeLa, Hep2G and SK-N-MC, HEK293) were used to monitor the influence of the four SNPs (rs945032, rs1799722, rs2528521 and rs509813) to investigate differences in expression that are possibly due to differences in TF expression in different cell types. BK-2 promoter SNPs rs945032 (genotype = GG) and rs1799722 (genotype = AA) showed approximately 40%-60% higher activity in HeLa cells as compared to their other homozygote alleles AA and GG, respectively (Figure 2). The BK-2 SNP rs945032 behaves in a reciprocal manner in two (HeLa, and HEK293) cell types. The BK-2 SNP rs1799722 allele 'C' increases expression only in HeLa while decreasing expression in the other three cell types, similar to CHRM1 marker rs509813. CT-R marker rs2528521 and CHRM1 marker rs509813 failed to show any influence on luciferase expression levels in HeLa and SK-N-MC cells. Finally, the CT-R SNP rs2528521, allele 'C', influences expression in a significant manner, but only in one cell line (Hep2G). Hence, different alleles behave differently in different cell environments.

## Discussion

SNPs that are located within coding regions and result in a change in the peptide sequence may be classified as 'damaging' or 'altering' if predicted to be in structurally or functionally important sites of the three dimensional structure of the protein. It is less straightforward to predict the functional importance of SNPs within regulatory regions. TFs tolerate variation in their binding sites and all positions in a site do not contribute equally to the binding energy. Therefore, the quantitative effect a given rSNP has on gene expression depends on its position and the bases involved. Complex human diseases like Parkinson's, diabetes and obesity are polygenic diseases, where many predisposing genetic and environmental factors together, over a period of time, cause a disease state. Differences in expression of genes and cellular concentrations of proteins due to common polymorphisms in 5' regulatory regions could equally elucidate gene function as the examination of non-synonymous SNPs in coding regions. We decided to test the 5'-FR of a group of physiologically and clinically important genes, for SNPs within TFBS, which could potentially influence the kinetics of binding affinity.

A common strategy for modeling the binding preferences of a transcriptions factor is to construct a position weight matrix (PWM) from known binding sites. PWMs are probabilistic models that capture the nucleotide preference at each position of a TFBS as well as the differential contribution of positions to the overall binding energy. When a putative binding site sequence is assessed using a PWM, a score is obtained that theoretically should be proportional to the binding energy between the TF and that sequence [10]. It has been convincingly shown, and generally accepted, that by considering DNA sequence conservation between mouse and human, the over-predictive nature of TFBS modeling can be significantly remedied. Therefore we chose for this work, not to include 'negative controls', that is, SNPs from outside of mouse-human conserved regions. We do indeed think that larger studies in the future should perhaps incorporate certain number of such negative controls to validate the theoretical predictions. Thus, using PWMs it should be possible to overcome the difficulties with rSNP detection stated above. We reasoned that if the score difference between alleles is large, it should correspond to a difference in gene expression that is reproducible in living cells. Using the JASPAR database of PWMs [27] and a phylogenetic footprinting strategy previously shown to be successful [17], we developed a method to detect putative TFBS and identify rSNPs likely to affect TF binding significantly. By incorporating phylogenetic footprinting, the method reported in this study emphasizes SNPs present in genomic regions that are highly conserved between human and mouse, thereby increasing the probability of a downstream functional influence of variations within theses sequences.

Electromobility Shift Assays (EMSA) produce DNA-protein binding interactions in artificial conditions. Therefore *in silico *prediction methods based on other *in vitro *or *in vivo *selection technologies, like 'systematic evolution of ligands by exponential enrichment' (SELEX), may not agree with the experimental outcome of EMSA procedures. Since the construction of PWMs is often concluded from published records based on SELEX enrichment approaches, it is informative to experimentally validate the predicted binding site using methods like EMSA. Therefore, we validated a subset of our predictions with *in vitro *electro-mobility shift. We used a stringent selection criterion, that is, qualified only alleles demonstrating an absolute binding score difference of 2.0 or more. A stringent selection criterion would no doubt decrease excessive hits and false positives at the expense of certain loss of true positives. Our results showed that approximately 60%-70% (i.e. 7 out of the selected 10 SNPs) of predicted sites (Table 2) bind proteins from HeLa nuclear extracts.

We finally attempted to correlate the EMSA findings from the 10 SNPs with influences on actual promoter activity within living cells. Due to mapping discrepancies of one SNP and failure to clone one promoter, we tested only four out of the seven EMSA-positive SNPs identified thus far. Of the four SNPs tested in a promoter-less expression vector, two (rs945032 and rs1799722) indicated significant influence on promoter activity, while two showed convincing and reproducible, yet comparatively limited influence (rs2528521 and rs509813) on promoter activity. The influence of these polymorphisms (rs945032 and rs1799722) indicate that any given functional variation within a regulatory region might exert a measurable influence within the context of a cell type determined by the TF expression profile of the cells and perhaps competitive binding of the TF to overlapping multiple binding sites.

There are several factors in the current approach which indicate that there are far more rSNPs than currently detected using available technologies. The EMSA assays employ HeLa nuclear extract, thereby limiting our findings to the TF expression repertoire of only HeLa nuclei. The TFBS package used a limited collection of high quality PWMs, which unfortunately represent only a small part of the approximately 2,000 known human and mouse TFs. The theoretical thresholds set for selections of alleles which are predicted to differentially bind TF require further rigorous testing to ensure that the present selection is optimal.

## Conclusions

From a total of approximately 200 SNPs in evolutionally conserved 5'-FR of 176 human GPCR genes, our prediction algorithm selected 36 SNPs with possible influence on TFBS. When ten of these 36 SNPs were tested for mobility shifts, seven exhibited a positive result, and four of these were further tested for influence on promoter activity using an *in situ *reporter system. Finally, two of the four showed significant and reproducible influences which were dependent on the cell environment. Thus starting from a large pool of potential regulatory SNPs, we successfully identified a small fraction that actually influenced promoter activity. We therefore propose a method for effective selection of functional, regulatory SNPs, in evolutionary conserved 5'-FR regions of human genes, as a means for identification of candidate SNPs for genetic association analysis studies.

## Methods

### Sequence alignment and TFBS detection

The GPCR genes were selected from Ensembl [25]. Human and mouse genome assemblies (versions hg12 and mm2, respectively) and mappings of GenBank and RefSeq cDNA sequences to the assemblies were retrieved from the UCSC Genome Browser Database [26]. In addition, cDNA sequences for the 176 7TM or GPCR genes (online supplement) were mapped to the human genome assembly and 50,821 mouse cDNA sequences from the RIKEN project [29] were mapped to the mouse genome assembly using the client/server version of BLAT [30] with default settings. For each of the 176 GPCR-encoding human cDNAs, we retrieved the genomic mapping with the highest number of matching bases. Orthologous mouse loci were identified by similarly retrieving mouse genomic mappings for mouse cDNAs defined as orthologs to the human cDNAs in GeneLynx [31]. To more reliably identify transcriptional start sites we searched for other cDNA mappings overlapping the retrieved mappings and indicating similar gene structures. For each gene, the cDNA mapping extending furthest 5' was then used for further analysis. We extracted human genomic sequences from -5000 to +100 relative to starts of human cDNA mappings and mouse genomic sequences from -30000 to +100 relative to starts of mouse cDNA mappings. Orthologous genomic sequences were aligned using BLASTZ [32] with default settings. Aligned regions preceding human cDNA mappings were searched for putative rSNPs as follows. SNP data for the human genomic regions was retrieved from dbSNP, build 114. For each SNP within an aligned region, two allelic versions of a 110-bp alignment slice centered around the SNP were searched for putative TFBS using the TFBS Perl modules [33] and all position-weight matrices in the JASPAR database describing vertebrate TFBS and having an information content of at least 7 [27]. Hits fulfilling the following 3 criteria were considered putative TFBS: (a) situated within regions of at least 70% sequence identity (conservation) over 50 base pairs; (b) situated at corresponding (aligned) positions in human and mouse sequences; (c) having a relative matrix score of at least 0.5 in both human and mouse sequences. Selected for further analysis were putative TFBS with a relative matrix score exceeding 0.8 in one of the human alleles and either undetected in the other allele or having a difference in absolute matrix score of at least 2 between the human alleles.

### Electromobility Shift Assays (EMSA)

Table 2 lists the sequences of oligonucleotides used for EMSA tests. For setting up of EMSA experimental procedures, an earlier published positive shift assay was reproduced using a polymorphism in the gene MMP12 [34]. Method modifications were then applied as described below.

Double stranded oligonucleotides were synthesized with 5'-GG dinucleotide overhangs. The 3'-end of the complementary strand was labeled with [α^32^P] dCTP with fill-in reaction using Klenow flagment. The labeled oligonucleotide were passed through ProbeQuant G-50 Micro Columns (Pfizer-Pharmacia Inc) and the concentration was adjusted to 0.035 nM. A 0.8 μl volume portion was mixed with 1.6 μl HeLa Nuclear Extract (Promega™), 1.6 μl 5x Gel Shift Binding 5x Buffer (Promega™), and 3.2 μl water. After 10 min incubation at room temperature, 0.8 μl of non-labeled competitor DNA, either one allele or the other allele, was added in varying concentrations (5-, 10-, 15-, 20- and 25-folds greater than the radio-labeled oligonucleotide). After 20 min room temperature incubation, the entire 8 μl reaction was loaded on polyaclylamide gel (5% 22.5 mM Tris/22.5 mM boric acid/0.5 mM EDTA buffer in, BIO RAD™). Thereafter, electrophoresis was performed in TBE for 20 min at 200 V. Gels were placed on Whatmann 3 MM™ filters and to facilitate drying a BIO RAD™ gel-dryer was used for 30 minutes. The dried gel were exposed to intensifying screen and analyzed by Typhoon Image Analyzer 9400 (Pfizer-Pharmacia Inc™).

### Sequencing and RFLP (Restriction fragment length polymorphism)

The frequency of Dopamine receptor D1 polymorphism rs267413 was determined by sequencing a 174 base-pair fragment of the promoter in 10 DNA samples from healthy Swedish individuals. The sequence of the forward primer used for PCR was 5'-GGGGTACCACTTGACCGTTCTGTTGCTTT-3' where a *KpnI *restriction site (GGTACC) and GG-dinucleotide was added to the 5' end. The sequence of the reverse primer was 5'-TCTTTTAAGCTCTACTGTGGGTGA-3'. Calcitonin receptor promoter polymorphism rs2528521 was analyzed by RFLP (fragment length was 334 bp, restriction enzyme *Tsp*45I). Forward primer sequence used for PCR was 5'-ACCCCCAAGGTGTCTCTTCT-3' and reverse primer: 5'- GAGGGACCCGAGTTAGACCT-3'. The primer sequences for Bradykinin promoter SNP rs1799722 were as follows: Forward primer 5'-CCAGGAGGCTGATGACGTCA-3'. The fourth base from 3'-end was changed from A to G from original genomic sequence to create a *Tsp45*I restriction site for RFLP analysis.

Reverse primer: 5'-TCAGTCGCTCCCTGGTACTG-3'. Fragment length amplified was 150 bp. PCR conditions for all RFLP and sequencing reactions were as follows: 94 C (4 min), followed by 42 cycles of 94 C (1 min), 61 C (30 sec) and 72 C (30 sec).

### Luciferase expression system for promoter activity analysis

A promoter-less luciferase vector (Basic PGL3, Promega™) was used for cloning known promoter regions between restriction sites *Kpn*I and *Bgl*II of the plasmid vector. Primers for CHRM1: Forward: 5'-GGGGTACCGCAGGACCCACATCTCTAGG-3' Reverse = 5'-GAAGATCTTCACCAGGGCACCCAAT-3'. Primers for BK-2: Forward = 5'-GGGGTACCATCTGAGACTCTGTTTCCC-3' reverse = 5'-GAAGATCTTTCAGTCGCTCCCTGGTACT-3'. Primers for CT-R: Forward = 5'-GGGGTACCCCTTGGAATCAACTTGCCT-3' reverse = 5'-TTCTCGAGCGTCCTTGGAATCAACTTGC-3'. Genomic DNA of 27 individuals of Nordic origin were amplified and sequenced to identify the genotype of sample DNA. Cloned DNA were sequenced by using primer set GLprimer2 : 5'-CTAGCAAATAGGCTGTCCC-3' and 5'-CTTTATGTTTTTGGCGTCTTCC-3'. HeLa cells were plated in 24 well plates one day before transfection in appropriate medium with serum without antibiotics. Basic PGL3 Plasmids containing the cloned promoter region (180 ng) were co-transfected with 20 ng of pRL-TK plasmid, using Lipofectamine 2000 (Invitrogen™). Luciferase activities were determined using a dual Luciferase Reporter Assay system (Promega™) according to the manufacturer's instructions.

## Abbreviations

TF: Transcription Factor; TFBS: transcription factor binding site; SNP: Single Nucleotide Polymorphism; GPCR: G-protein coupled receptors; 5'-FR: 5' flanking regulatory region.

## Authors' contributions

Original Concepts and supervision: CW, WWW, BL and SM-T; Bioinformatics: PGE, BL and SMT; Running costs: SMT and CW; Manus preparation: SMT, PGE, CW; Electromobility Shift Assays and RFLP assays: YM; Luciferase expression: MAF and YM.

## Supplementary Material

Additional File 1 ENSG ids A list of 176 initial GPCRs considered for this study, along with the Ensembl ENSG Ids. Click here for file

Additional File 2 Alignments Alignment information for sequence flanking rs945032 and rs1799722 in human and mouse. Click here for file
